# 腺癌胸水标本中PD-L1的蛋白表达与临床病理特征及分子改变的相关性研究

**DOI:** 10.3779/j.issn.1009-3419.2020.03.03

**Published:** 2020-03-20

**Authors:** 海玥 马, 佳 贾, 会芹 郭, 焕 赵, 聪 王, 琳琳 赵, 悦 孙, 卫华 李, 智慧 张

**Affiliations:** 100020 北京，国家癌症中心/国家肿瘤临床医学研究中心/中国医学科学院北京协和医学院肿瘤医院病理科细胞学室 Cytology Section, Department of Pathology, National Cancer Center/National Clinical Research Center for Cancer/Cancer Hospital, Chinese Academy of Medical Sciences and Peking Union Medical College, Beijing 100020, China

**Keywords:** 程序性死亡配体, 免疫细胞化学, 肺腺癌, 胸水细胞块, 二代测序, Programmed death ligand 1, Immunocytochemistry, Lung adenocarcinoma, Pleural effusion cell block, Next-generation sequencing

## Abstract

**背景与目的:**

非小细胞肺癌的免疫治疗药物pembrolizumab需要基于包括免疫组织化学（immunohistochemistry, IHC）在内的检测结果，即通过检测程序性死亡配体1（programmed cell death protein 1, PD-L1）的表达等手段来预测治疗反应。评估肺腺癌细胞学标本免疫细胞化学（immunocytochemistry, ICC）方法检测PD-L1的可行性，并探讨PD-L1表达与临床病理及分子特征的相关性。

**方法:**

收集60例肺腺癌胸水细胞学标本应用PD-L1 sp263试剂按照生产说明书进行免疫细胞化学染色，同时对胸水细胞学标本做高通量二代测序（next-generation sequencing, NGS），探讨PD-L1与驱动基因突变的相关性。

**结果:**

60例肺腺癌胸水细胞块标本ICC检测中，有35例PD-L1表达阳性，阳性表达率为58.3%。本院57例组织学标本PD-L1 IHC表达的阳性率为33.3%，细胞学标本与组织学标本差异无统计学意义（*P* > 0.05）。60例细胞学标本中26例接受NGS检测，15例（57.7%）发现表皮生长因子受体（epidermal growth factor receptor, EGFR）突变，经统计PD-L1表达与EGFR突变未发现相关性。PD-L1的阳性表达率与研究人群的年龄、性别、是否淋巴结或远处转移及是否进行放化疗或靶向治疗均未发现相关性（*P* > 0.05）。

**结论:**

在无手术标本可取时，胸水细胞学细胞块标本可以对PD-L1进行免疫细胞化学检测，其结果具有可行性。

## Abstract (en)

**Background and objective:**

Pembrolizumab, an immunotherapy for advanced non-small cell lung cancer (NSCLC), needs to predict treatment response based on test results including immunohistochemistry (IHC), which detects the expression of programmed death ligand 1 (PD-L1). The aim of this study was to evaluate the feasibility of immunocytochemistry (ICC) in NSCLC cytology to detect PD-L1 and to investigate the correlation between PD-L1 expression and clinical pathology and molecular features.

**Methods:**

Sixty cases of lung adenocarcinoma pleural cytology were collected and PD-L1 sp263 reagent was used for immunocytochemical staining according to the manufacturer's instructions. Next-generation sequencing (NGS) was performed on pleural cytology specimens to explore its correlation.

**Results:**

Of the 60 cases of lung adenocarcinoma pleural effusion cell block, 35 cases were positive for PD-L1 expression, and the positive expression rate was 58.3%. The positive rate of PD-L1 expression in the specimens of our hospital was 33.3%, and there was no significant difference between the cytological specimens and the histological specimens (*P* > 0.05). Of the 60 cytological specimens, 26 were tested for NGS, and 15 (57.7%) were found to have epidermal growth factor receptor (EGFR) mutations. No correlation was found between PD-L1 expression and EGFR mutation. The positive expression rate of PD-L1 were not correlated with the age and gender in the study population (*P* > 0.05).

**Conclusion:**

When no surgical specimens are available, pleural cytology cell block specimens can be used for immunocytochemical detection of PD-L1, and the results are feasible.

目前，肺癌是世界上发病率及死亡率均位居第一位的癌症，且发病率逐年增高，其中非小细胞肺癌（non-small cell lung cancer, NSCLC）占85%^[1]^。近年来，免疫治疗取得了长足的进展，肿瘤诱导的特异性T细胞活化抑制，主要由抑制途径介导，为程序性死亡蛋白受体1（programmed cell death protein 1, PD-1）或其配体程序性死亡配体1（programmed cell death ligand 1, PD-L1）相互作用的特异性抗体开辟了一种全新的治疗选择。在黑色素瘤、肺癌等多个瘤种的治疗过程中取得了显著疗效，已被美国食品药品监督管理局批准用于临床治疗。目前PD-L1的检测可以依赖组织学标本，但是部分肺腺癌患者就诊时已经处于晚期，发生远处转移，文献[2]报道大约50%以上肺癌患者随着病情进展出现转移，约15%患者初诊时已有胸腔积液，浆膜腔积液是可获得的形态学标本来源之一。目前免疫组织化学（immunohistochemistry, IHC）方法是一种重要的PD-L1检测方法，免疫组织化学方法在细胞学标本中的应用已经有许多报道，尤其应用细胞块标本的检测也已经成熟。应用转移灶的细胞学标本制作成细胞块对PD-L1进行免疫细胞化学检测，其结果能否应用于临床诊断中，目前国内还未见报道。

本研究收集肺腺癌恶性胸水标本，制作成细胞块进行PD-L1免疫细胞化学检测，分析比较细胞块石蜡切片和组织学检测结果的一致性和差异性，以及细胞块石蜡切片PD-L1表达与肺癌驱动基因表达的相关性，探索细胞学标本PD-L1检测的可行性。

## 1 材料与方法

### 1.1 标本收集

选取2017年11月-2018年10月于中国医学科学院肿瘤医院就诊的患者，纳入标准：①胸水细胞学标本；②细胞学诊断为肺腺癌，且经免疫细胞化学证实；③肿瘤细胞含量 > 30%；④肿瘤细胞数 > 100个。共收集符合条件的恶性胸水患者60例，其中男性28例，女性32例，中位年龄为55岁，共51例有影像学资料及完整治疗过程。排除标准：肿瘤细胞数量≤100个的患者及病例资料不完整者。

### 1.2 标本的制备

将新鲜的胸腔积液静置20 min-30 min，收集底部的沉淀液，将其放置在离心管中，离心处理10 min，转速为2, 500 r/min，倾其上清液后，加入少许血浆（中国医学科学院肿瘤医院血库提供），后滴入相应数量的凝血酶（长春雷允上药业有限公司生产），快速搅拌形成凝块。将凝块置于贴有标识的包埋盒后于10%中性福尔马林液中固定。依次进行脱水、包埋、切片。切片两张，一张用于PD-L1染色，一张用于阴性对照。

### 1.3 免疫细胞化学

#### 1.3.1 试剂与仪器

一抗为即用型SP263（Roche，罗氏公司，美国），二抗为罗氏OptiView DAB显色系统。缓冲液、清洗液、免疫组化抗原修复液、苏木素染色液和返蓝染色液均为罗氏全自动免疫组化仪的配套产品。BenchMark GX全自动免疫组化仪为美国罗氏公司产品。

#### 1.3.2 染色方法

将所需检测试剂和辅助试剂放置在全自动免疫组化仪试剂架上。将固定后的切片根据抗体的修复时间、孵育时间及温度等不同在电脑中设定相应的染色方案。通过电脑输入一抗名称，Ebar条码打印机打出标签，分别贴在相应玻片上。将玻片放入罗氏BenchMark GX全自动免疫组化仪的相应位置，其余步骤均由机器自动操作。每组加入1张胎盘切片作为阳性对照。染色完成后取出玻片，用清洗液洗去缓冲液，经95%乙醇和二甲苯脱水透明，封片。

### 1.4 高通量测序（next-generation sequencing, NGS）检测

本实验使用基于杂交捕获法的高通量NGS方法，对NSCLC相关肿瘤驱动基因进行基因变异检测，包括表皮生长因子受体（epidermal growth factor receptor, *EGFR*）、Kirsten鼠肉瘤病毒癌基因（Kirsten rat sarcoma viral oncogene homolog, *KRAS*）、间变性淋巴瘤激酶（anaplastic lymphoma kinase, *ALK*）、*ROS1*（c-ros oncogene 1 receptor tyrosine kinase）、间质-上皮转化因子（mesenchymal-epithelial transition factor, *MET*）、鼠类肉瘤滤过性毒菌致癌同源体B1（v-raf murine sarcoma viral oncogene homolog B1, *BRAF*），检测的基因变异类型包括基因的点突变、插入及缺失、扩增，另外还包括*ALK*、*ROS1*的融合状态。检测平台为Illumina NextSeq 50操作流程按照说明书进行，流程同参考文献^[3]^。

### 1.5 免疫细胞化学结果评价

免疫细胞化学染色后，在显微镜下评价PD-L1染色得分。肿瘤比例评分（tumor proportion score, TPS）指肿瘤细胞中染色阳性的细胞比例。本实验将1%≤TPS < 50%，TPS≥50%作为阳性表达区间进行分析^[4]^。由两名有经验的细胞病理医师独立判读，任何不一致的结果均重新评价得到共识。

### 1.6 统计学方法

采用SPSS 23.0统计软件包进行统计学处理，计数资料行χ^2^检验，以*P* < 0.05为差异有统计学意义。

## 2 结果

60例胸水细胞学肺腺癌标本，PD-L1阳性表达的有35例，阳性表达率为58.3%；肿瘤细胞PD-L1的阳性表达数目1%-49%为20例，阳性率为33.3%；肿瘤细胞PD-L1的阳性表达数目≥50%为15例，阳性率为25.0%，见图 1。60例标本的临床特征见表 1，由表 1可见，PD-L1的阳性表达率与研究人群的年龄、性别、是否存在淋巴结转移、是否存在远处转移及是否进行放化疗或靶向治疗无相关性（*P* > 0.05）。

**1:**
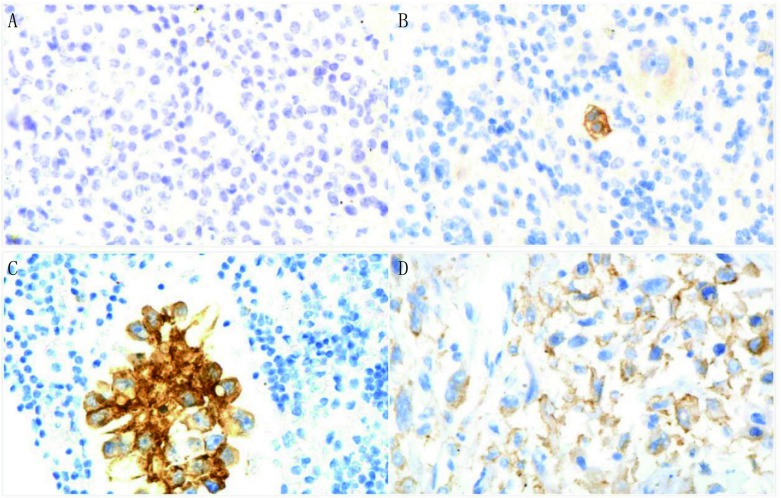
PD-L1在细胞学及组织学标本中的IHC表达结果。A：PD-L1免疫细胞化学表达结果呈阴性（DAB染色，×400）；B：PD-L1免疫细胞化学表达结果呈阳性，阳性肿瘤细胞数目 < 50%（DAB染色，×400）；C：PD-L1免疫细胞化学表达结果呈强阳性，阳性肿瘤细胞数目≥50%（DAB染色，×400）；D：手术后组织学标本，PD-L1免疫组织化学表达结果呈阳性（DAB染色，×400） PD-L1 IHC expression in cytology and surgical specimens. A: 1 representative cytology case with negative PD-L1 expression (DAB staining, ×400); B: 1 representative cytology case with positive PD-L1 expression (≥1% but < 50%) (DAB staining, ×400); C: 1 representative cytology case with high PD-L1 expression (≥50%) (DAB staining, ×400); D: PD-L1 expression positive in representative surgical specimens (DAB staining, ×400). IHC: immunohistochemistry

**1:** PD-L1的表达与患者临床特征的关系 The relationship between the expression of PD-L1 and the clinicopathological features of the patients

Clinicopathological features	*n*	Positive rate	*P*
Gender			0.484
Male	28	53.6% (15/28)	
Female	32	62.5% (20/32)	
Age (yr)			0.432
≥55	30	53.3% (16/30)	
< 55	30	63.3% (19/30)	
Lymphatic metastasis			0.070
Yes	36	61.1% (22/36)	
No	15	33.3% (5/15)	
Distant metastasis			0.369
Yes	33	57.6% (19/33)	
No	18	44.4% (8/18)	
Radiotherapy or chemotherapy or targeted therapy			0.361
Yes	20	45.0% (9/20)	
No	31	58.1% (18/31)	
PD-L1: programmed cell death ligand 1.			

本院应用术后组织学标本57例，抗体为sp263检测PD-L1的表达，有19例表达阳性，阳性表达率为33.3%，见图 1。

60例患者中26例用高通量杂交捕获法同时进行了NGS，其中21例（80.1%）有遗传变异。发现12例PD-L1表达阳性的患者中，有5例发生了*EGFR*基因突变，其中第19号外显子突变3例，第21号外显子突变2例，PD-L1表达与*EGFR*基因突变无相关性（*P*=0.233 > 0.05），见图 2。另外14例PD-L1表达阴性的病例中，有10例发生了*EGFR*基因突变，其中第19号外显子突变5例，第21号外显子突变5例。*KRAS*及*MET*基因突变例数各3例，PD-L1全部表达阳性，而且表达均为TPS≥50%强阳性。2例*BRAF*突变的患者，PD-L1表达均为阴性，2例*ROS1*基因融合患者中，PD-L1表达阴性及阳性各1例。3例*ALK*基因融合患者中，有PD-L1阳性表达2例。*KRAS*、*ROS1*、*BRAF*、*MET*及*ALK*基因异常由于病变例数少，无法进行统计学处理。*EGFR*、*KRAS*、*ROS1*、*BRAF*、*MET*及*ALK*基因异常情况与PD-L1表达关系见表 2。

**2:**
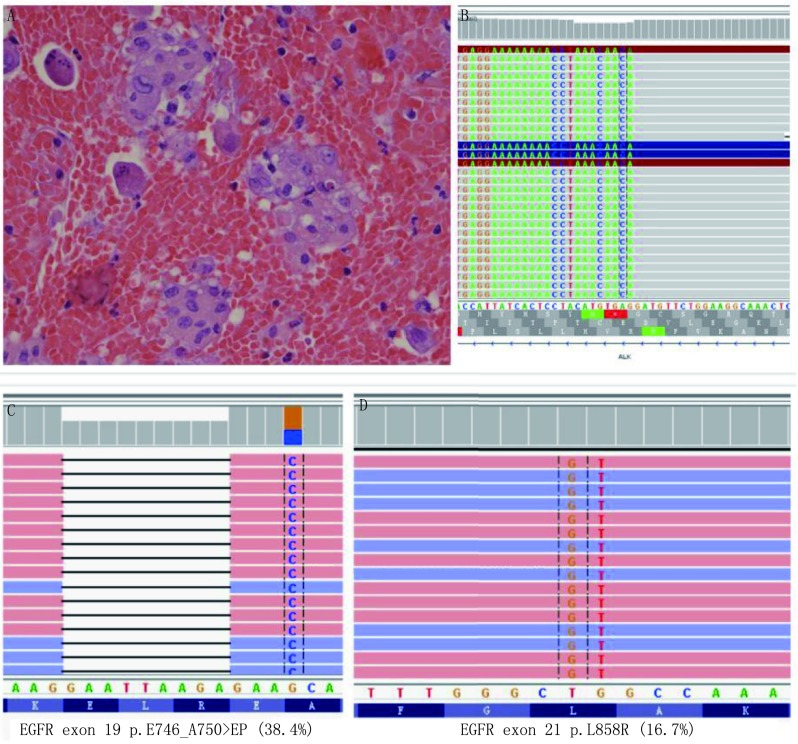
胸水细胞块HE染色及高通量二代测序突变结果。A：胸水细胞块HE染色（×400）；B：*ALK*基因的高通量二代测序突变结果；C：*EGFR*基因的高通量二代测序第19号外显子突变结果；D：*EGFR*基因的高通量二代测序第21号外显子突变结果 HE staining of pleural effusion cell block and mutation results by NGS. A: pleural effusion cell block (HE staining, ×400); B: 1 representative cytology case with *ALK* translocations by NGS; C: 1 representative cytology case with *EGFR* exon 19 mutations by NGS; D: 1 representative cytology case with *EGFR* exon 21 mutations by NGS. EGFR: epidermal growth factor receptor; NGS: next-generation sequencing; ALK: anaplastic lymphoma kinase

**2:** PD-L1表达与NGS检测中基因异常结果的关系 The relationship between the expression of PD-L1 and the molecular alterations of NGS

Molecular alterations	PD-L1 positive	PD-L1 negative
*EGFR* exon 19	3	5
*EGFR* exon 21	2	5
*KRAS*	3	0
*ROS1*	1	1
*BRAF*	0	2
*MET*	3	0
*ALK*	2	1
KRAS: Kirsten rat sarcoma viral oncogene homolog; ROS1: c-ros oncogene 1 receptor tyrosine kinase; BRAF: v-raf murine sarcoma viral oncogene homolog B1; MET: mesenchymal-epithelial transition factor.		

## 3 讨论

采用TPS方法评价得分，本研究肺腺癌胸水细胞块组中免疫细胞化学PD-L1检测总阳性率为58.3%，应用同种抗体美国罗氏SP263，本院组织学标本的一组数据显示肺腺癌PD-L1总阳性率为33.3%^[5]^。两组数据无统计学差异（*P*=0.07 > 0.05）。但胸水细胞块标本的阳性率明显高于手术后组织学标本，其结果与文献[6]报道相符。Heymann等^[6]^报道，在40例细胞学标本和76例组织学标本非一对一PD-L1免疫组织化学检测中，阳性表达率分别为39%、25%（*P*=0.083），与72例小活检标本的阳性表达率比较无明显差异（39% *vs* 23%; *P*=0.094）。同样，Bratton等^[7]^也有相似的研究结果，他们对12例腺癌细胞学标本和12例腺癌组织学标本非一对一PD-L1免疫组织化学检测，其阳性表达率分别为67%、50%。Noll等^[8]^研究表明41例细胞学涂片或细胞块与同例组织学标本一对一PD-L1免疫组织化学检测，发现仅有1例标本两者结果不一致，细胞块表达阳性，而组织学标本表达为阴性。Rehman等^[9]^报道，在同一个实验室用同一型号的抗体在同一瘤体内不同蜡块的切片中PD-L1检测出现不同的表达结果，由此表明同一瘤体内存在着细胞的异质性。

细胞学标本比组织学标本PD-L1免疫组织化学阳性表达率高的另一个原因可能是原发灶和转移灶问题^[10]^。胸腹水标本来自肺腺癌晚期患者发生广泛转移的转移灶标本，组织学标本大多来源于手术患者的原发灶，不能确定是否PD-L1表达的肿瘤细胞恶性程度更高，更具有侵袭性，更容易发生远处转移，导致几项研究结果^[6-8]^均表明，细胞学标本PD-L1检测的阳性表达率高于组织学标本。但采集标本间隔时间长短是否影响PD-L1表达，以及放化疗、靶向治疗等治疗手段是否会造成同一患者不同时期标本表达的差异还有待研究。

本研究有26例用高通量二代测序杂交捕获法同时检测了*EGFR*、*KRAS*、*ROS1*、*BRAF*、*MET*及*ALK*基因异常情况，15例有*EGFR*基因突变的病例中有5例PD-L1表达阳性，PD-L1表达与*EGFR*基因突变无相关性（*P*=0.233 > 0.05）。同时也未发现*EGFR*基因第19号外显子突变与第21号外显子突变的患者与PD-L1表达存在差异。本研究中虽然*KRAS*及*MET*基因突变的病例PD-L1表达均为阳性，但是KRAS仅3例，MET仅3例，由于病变例数少，无法进行统计。美国的研究^[11]^表明PD-L1表达与*KRAS*突变具有显著相关性，但是与其他突变基因未见相关性。他们发现190例接受NGS基因检测的病例中74例（38.9%）伴有遗传变异，其中*KRAS*突变56例，*KRAS*突变率为29.5%。他们的结果显示有遗传改变的病例PD-L1阳性表达的病例数明显高于无遗传改变的病例数。PD-L1表达水平仅在*KRAS*突变组和无*KRAS*突变组之间有显著差异（78% *vs* 40%）。目前，美国食品和药物管理局已批准免疫调节疗法作为晚期NSCLC患者的二线药物，但是对于无*EGFR*突变或*ALK*重排的患者当PD-L1表达 > 50%时可以作为NSCLC患者的一线药物应用于临床^[12]^。基于PD-L1表达与PD-1/PD-L1抑制剂临床疗效具有显著相关性的数据，2018年美国国立综合癌症网络（National Comprehensive Cancer Network, NCCN）指南已经将PD-L1列为NSCLC患者的推荐常规检测对象，建议结合PD-L1表达状态进行治疗选择^[13]^。肿瘤细胞PD-L1的表达目前被认为是抗PD-1/PD-L1治疗的优势人群选择的最合理标志物，PD-1/PD-L1抑制剂的疗效与PD-L1表达水平密切相关，PD-L1表达水平越高，治疗有效的机会将大大增加，但目前检测PD-L1表达所采用的检测平台、抗体、评价方法的体系均有所不同，使得PD-L1检测存在缺乏一致性与检测金标准的缺陷，仍需进一步探索。由于细胞学标本的敏感性高，可能部分组织学标本PD-L1表达阴性患者也可能在转移灶的细胞学标本中有PD-L1的表达，可以应用PD-L1免疫抑制剂进行治疗。因此，细胞学标本的高敏感性更加有助于晚期肺腺癌患者从PD-L1抑制剂的治疗中获益。

总之，肺腺癌胸水细胞学标本可以协助PD-L1的免疫细胞化学检测，有助于肺腺癌晚期患者应用PD-L1抑制剂进行治疗。

## Author contributions

Zhang ZH conceived and designed the study. Ma HY and Jia J performed the experiments. Ma HY and Jia J analyzed the data. Wang C, Zhao LL, Sun Y and Li WH contributed analysis tools. Guo HQ and Zhao H provided critical inputs on design, analysis, and interpretation of the study. All the authors had access to the data. All authors read and approved the final manuscript as submitted.

## References

[1] Bray F, Ferlay J, Soerjomataram I (2018). Global cancer statistics 2018: GLOBOCAN estimates of incidence and mortality worldwide for 36 cancers in 185 countries. CA Cancer J Clin.

[2] Tian Y, Wang Y, Liu SY (2016). Detection and clinical significance of EGFR mutations in lung cancer patients with malignant pleural effusion. Zhongguo Yi Ke Da Xue Xue Bao.

[3] Li W, Zhang J, Guo L (2017). Combinational analysis of FISH and immunohistochemistry reveals rare genomic events in ALK fusion patterns in NSCLC that responds to crizotinib treatment. J Thorac Oncol.

[4] Munari E, Rossi G, Zamboni G (2018). PD-L1 assays 22C3 and SP263 are not interchangeable in non-small cell lung cancer when considering clinically relevant cutoffs: an interclone evaluation by differently trained pathologists. Am J Surg Pathol.

[5] Yuan P, Guo CY, Li Y (2018). Consistency of PD-L1 immunohistochemical detection platforms in biopsy samples with advanced lung adenocarcinoma: a multicenter study. Zhonghua Bing Li Xue Za Zhi.

[6] Heymann JJ, Bulman WA, Swinarski D (2017). PD-L1 expression in non-small cell lung carcinoma: comparison among cytology, small biopsy, and surgical resection specimens. Cancer Cytopathol.

[7] Bratton L, Russell D, Yong Q (2016). Comparison of PD-L1 immunostaining for non-small cell carcinoma of the lung between paired cytological and surgical specimens. J Am Soc Cytopathol.

[8] Noll B, Wang WL, Gong Y (2018). Programmed death ligand 1 testing in non-small cell lung carcinoma cytology cell block and aspirate smear preparations. Cancer Cytopathol.

[9] Rehman JA, Han G, Carvajal-Hausdorf DE (2017). Quantitative and pathologist-read comparison of the heterogeneity of programmed death-ligand 1 (PD-L1) expression in non-small cell lung cancer. Mod Pathol.

[10] Mansfield AS, Aubry MC, Moser JC (2016). Temporal and spatial discordance of programmed cell death-ligand 1 expression and lymphocyte tumor infiltration between paired primary lesions and brain metastases in lung cancer. Ann Oncol.

[11] Mei P, Shilo K, Wei L (2019). Programmed cell death ligand 1 expression in cytologic and surgical non-small cell lung carcinoma specimens from a single institution: association with clinicopathologic features and molecular alterations. Cancer Cytopathol.

[12] Reck M, Rabe KF (2017). Precision diagnosis and treatment for advanced non-small-cell lung cancer. N Engl J Med.

[13] Ettinger DS, Wood DS, Aisner DL (2014). NCCN clinical practice guidelines in oncology: non small cell lung cancer. version 1. J Natl Compr Canc Netw.

